# Supplementary material for: Random Matrix Theory Analysis of a Temperature-Related Transformation in Statistics of Fano–Feshbach Resonances in Thulium Atoms
Source: Entropy (Basel). 2020 Dec 10;22(12):1394. doi: 10.3390/e22121394 (PMC7763200; doi:10.3390/e22121394)
Supplement: Supplementary file 1 [file entropy-22-01394-s001.pdf]

Supplementary Material for Random matrix theory analysis of a temperature-related transformation in statistics of Fano-Feshbach resonances in Thulium atoms

E.T. Davletov[1,2], V.V. Tsyganok[1,2,3], V. A. Khlebnikov[1], D. A. Pershin[1,4], A.V. Akimov[1,4,5]

[1] Russian Quantum Center, Business Center "Ural", 100A Novaya Street Skolkovo, Moscow, 143025, Russia
[2] Moscow Institute of Physics and Technology, Institutskii pereulok 9, Dolgoprudny, Moscow Region 141701, Russia
[3] National University of Science and Technology MISIS, Leninsky Prospekt 4, Moscow, 119049, Russia,
[4] PN Lebedev Institute RAS, Leninsky Prospekt 53, Moscow, 119991, Russia
[5] Texas A&M University, TAMU 4242, College Station, TX 77843, USA
* Correspondence: akimov@physics.tamu. edu;

The stark shift

The Stark energy shift is given by:

$$\begin{aligned}
U(\omega) &= -\frac{1}{2\varepsilon_0 c} I(r)\,\mathrm{Re}[\alpha_{tot}] = U_s + U_\upsilon + U_t \\
U_s &= -\frac{1}{2\varepsilon_0 c} I(r)\,\mathrm{Re}[\alpha_s(\omega)] \\
U_\upsilon &= -\frac{1}{2\varepsilon_0 c} I(r)\,\varepsilon\cos\theta_k \frac{m_F}{2F}\mathrm{Re}[\alpha_\upsilon(\omega)] \\
U_t &= -\frac{1}{2\varepsilon_0 c} I(r)\frac{3m_F^2 - F(F+1)}{F(2F-1)}\cdot\frac{3\cos^2\theta_p - 1}{2}\mathrm{Re}[\alpha_t(\omega)]
\end{aligned} \quad . \quad (1)$$

Here, ω is trapping light frequency; ε_0 the vacuum permittivity; c the speed of light; $I(r)$ the laser intensity profile; $\varepsilon = \left|\vec{u}^* \times \vec{u}\right|$ the ellipticity parameter with $\vec{u}$ as the normalized Jones vector; $\theta_p = \angle\left(\vec{E},\vec{B}\right)$ and $\theta_k = \angle\left(\vec{k},\vec{B}\right)$; $\vec{E}$ and $\vec{B}$ are the electrical and magnetic components of the trapping beam light with the wave vector $\vec{k}$; α_{tot} is the total atomic polarizability; and $\alpha_s(\omega)$, $\alpha_\upsilon(\omega)$, $\alpha_t(\omega)$ are scalar, vector, and tensor dynamic dipole polarizabilities, respectively. A more detailed description of these quantities and trap geometry can be found in[21].
